# Evaluation of Targeted Mass Cholera Vaccination Strategies in Bangladesh: A Demonstration of a New Cost-Effectiveness Calculator

**DOI:** 10.4269/ajtmh.14-0159

**Published:** 2014-12-03

**Authors:** Christopher Troeger, David A. Sack, Dennis L. Chao

**Affiliations:** Center for Statistics and Quantitative Infectious Diseases, Vaccine and Infectious Disease Division, Fred Hutchinson Cancer Research Center, Seattle, Washington; Department of International Health, Johns Hopkins Bloomberg School of Public Health, Baltimore, Maryland

## Abstract

Growing interest in mass vaccination with oral cholera vaccine in endemic and epidemic settings will require policymakers to evaluate how to allocate these vaccines in the most efficient manner. Because cholera, when treated properly, has a low case fatality rate, it may not be economically feasible to vaccinate an entire population. Using a new publicly available calculator for estimating the cost-effectiveness of mass vaccination, we show how targeting high-risk subpopulations for vaccination could be cost-effective in Bangladesh. The approach described here is general enough to adapt to different settings or to other vaccine-preventable diseases.

## Introduction

Cholera is an ancient disease, first formally described to the Western world by British physicians in 18th century colonial India but known to the peoples of the Ganges Delta in South Asia for much longer.^1^ Today, cholera is a problem in populations that lack access to safe water and sanitation, and has established itself as a major burden of disease outside of South Asia, notably in Africa and Hispaniola.^2^ The increasing availability of and demand for an oral cholera vaccine (OCV) suggests that an integrated strategy that incorporates OCV is a desirable option for reducing the burden of disease in many endemic and epidemic settings.^3^–^8^ As cholera primarily affects the developing world where economic resources are limited, there is often a dilemma of how to allocate these vaccines in the most efficient manner.^3^–^6^,^8^–^10^

Economic analyses are often used to guide policy decisions regarding the most efficient use of resources.^11^–^16^ Cost-effectiveness analyses have become popular in health and development, notably the World Health Organization's (WHO) Choosing Interventions that are Cost-effective (CHOICE) Project, and are an important factor for governments and other decision makers when considering how to best allocate limited resources and to assess the value of new vaccines such as OCV.^17^–^20^ Here, we present an application of a newly developed, publicly available tool for analyzing the cost-effectiveness of cholera mass vaccination.

We use the cost-effectiveness tool to explore the cost-effectiveness of targeting high-risk populations for cholera vaccination in Bangladesh. Bangladesh was selected as a case study because of its long history of cholera, the presence of ongoing cholera surveillance, and its potential interest in introducing oral cholera vaccine as part of a national cholera prevention and control strategy.^21^ Although this report focuses on cholera in Bangladesh, the methods used here are general enough to apply to other populations and other vaccine-preventable diseases.

## Methods

We have developed a tool, the *Vaccine Introduction Cost-Effectiveness* (VICE) calculator, to investigate the cost-effectiveness of targeting different sub-populations in Bangladesh for cholera vaccination. The calculator computes cost-effectiveness outcomes. The VICE calculator is implemented as a spreadsheet in Microsoft Excel (Microsoft Corporation, Redmond, WA, 2011) and is available for download from http://stopcholera.org. Users have full control over parameters to describe the epidemiology of a population, vaccine characteristics, and economic values.

### Economic analysis.

Typically, economic analyses of health interventions compare current practices and prospective new interventions, defined in this work as no vaccination and vaccination, respectively.^22^ This analysis takes a societal financial perspective. The vaccination costs are borne by the public sector and the costs of illness averted consider both direct (medical and non-medical) and indirect (such as lost wages) costs.^21^,^23^ We have chosen to use disability-adjusted life years (DALYs) to be consistent with the prevalent literature in developing country and cholera vaccine contexts and the WHO CHOICE program.^11^,^14^,^15^ Many cost-effectiveness ratios express Cost/DALY averted; the calculation is described in five equations below, adapted from previous work on OCV cost-effectiveness^24^:

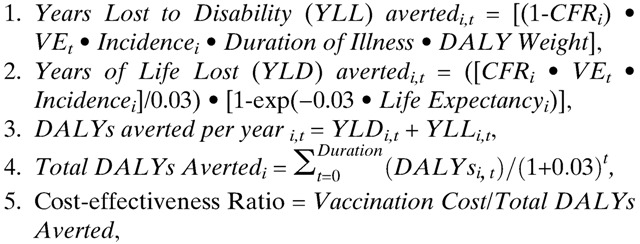

where VE is the efficacy of the vaccine for preventing infection, the DALY weight is an estimate of disability caused by disease, CFR is the case fatality ratio, *t* is the time in years, and *i* indicates the subpopulation *i*. The vaccine efficacy is at an individual level, therefore the cost per DALY averted does not depend on vaccine coverage.

The cost-effectiveness measures can be computed for a single, homogeneous population or for separate components of a heterogeneous population that consists of subpopulations or strata with differences in disease incidence, case fatality rate, life expectancy, or vaccine efficacy. We explore four scenarios for vaccination strategy to illustrate subpopulation targeting^19^,^25^:

1. Untargeted, non-selective: mass vaccination.
2. Age targeted: preferentially vaccinating children (4 age groups: 1–4 years, 5–9 years, 10–14 years, 15 years and older).
3. Geographic targeted: vaccinating areas at elevated risk.
4. Targeting populations with poor access to treatment: vaccinating people with a higher case fatality ratio.

According to WHO convention, the ratio of Program Cost to DALYs Averted in a cost-effectiveness analysis is classified by the per capita national gross domestic product (GDP) of the country of interest: < GDP/capita classifies an intervention as “very cost-effective”; between 1 and 3 times GDP/capita is “cost-effective”; and > 3 times the GDP/capita is “cost-ineffective.”^17^ These threshold guidelines have been used in this analysis.

### Data and parameters.

The values used for the cost-effectiveness calculations, including the demography and epidemiology of cholera in Bangladesh, are provided in Table 1. The incidence of cholera in this analysis comes from passive, clinic-based surveillance, therefore the observed incidence is likely an underestimate of the true burden of cholera.^38^ Children < 1 year of age are not considered as OCV in this analysis, is not currently licensed for use in this age group. Recent work from Dhaka, Bangladesh, and Kolkata, India has provided region-specific estimates of vaccine effectiveness, duration of vaccine-derived immunity, and the cost of infection for the use of *Shanchol*, a WHO prequalified OCV.^23^,^28^ The cost of vaccination includes purchasing and delivery costs, which does not take economies of scale into account. The duration of illness, the disability weight (applying only to the duration of illness), and discounting rate were taken from the literature.^22^,^24^,^36^

### Mathematical model of cholera transmission.

A mathematical model of cholera transmission in a population in rural Bangladesh was used to estimate the direct and indirect protection from mass cholera vaccination in an analysis supplemental to the main results. Indirect protection from mass vaccination, sometimes known as “herd protection,” can increase cost-effectiveness estimates.^39^ Because predicting the indirect protection from mass vaccination is difficult and does not generalize to different epidemic settings, only direct protection from OCV is considered in the VICE calculator.

To estimate the overall effect of mass cholera vaccination, we used a stochastic mathematical model of cholera transmission described in Longini and others, 2007.^40^ This agent-based model simulated the spread of cholera for one season in a synthetic population based on demographic information from MATLAB, Bangladesh. Cholera transmission within the population was based on a susceptible-exposed-infectious-recovered (SEIR) framework. The model was calibrated using surveillance data from a mass cholera vaccination trial.^41^

We ran the model for a single simulated year, 100 times for each of several levels of vaccine coverage of the target population (those 1 year of age and older), from 0% to 100%. Only individuals 1 year of age and older were eligible for vaccination. We defined the fraction of cases averted for a given coverage level to be one minus the ratio of the average illness attack rate for that level of vaccine coverage to the attack rate with no vaccination. We compared the fraction of cases averted to what one would expect from direct protection only, which is vaccine coverage times vaccine efficacy (65%, see Table 1).

## Results

We evaluated the cost-effectiveness of different mass cholera vaccination strategies in Bangladesh. We assume that the vaccine has a 65% direct, protective efficacy for preventing infection and lasts for 3 years unless otherwise stated.^28^ The parameters used in the analyses are summarized in Table 1. Table 2 summarizes the primary economic and health outcomes for each of the vaccination strategies that do and do not prioritize various high-risk populations.

### Non-selective mass vaccination.

**Figure 1.**
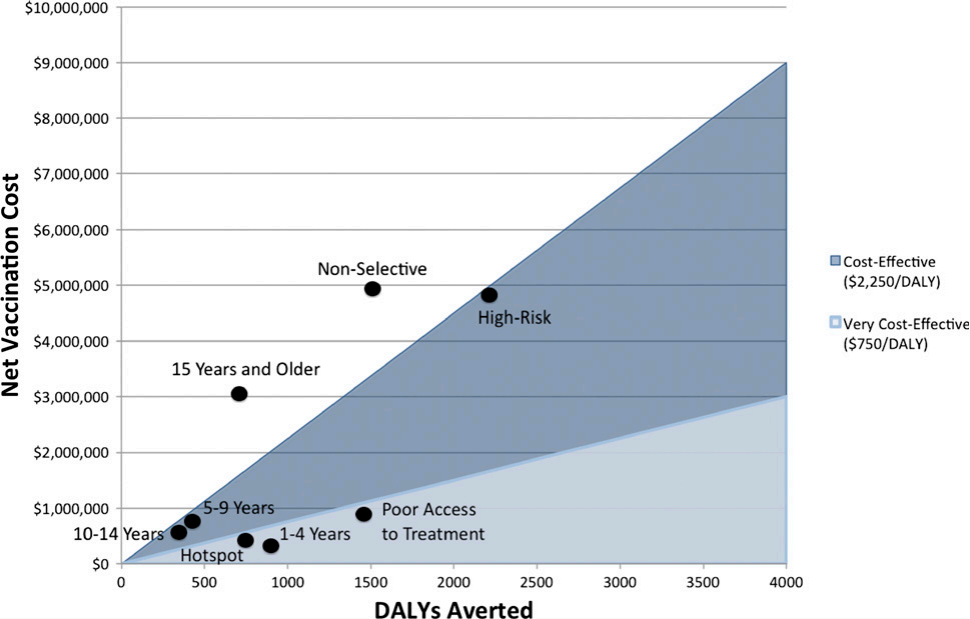
Economic assessment of non-selective and targeted mass cholera vaccination in Bangladesh. Each point represents the total cost of vaccinating a population or subpopulation and the expected number of disability-adjusted life years (DALYs) averted from vaccination. Vaccination cost is based on hypothetical fully vaccinated populations of 1,000,000 individuals (100% coverage). The age-based subpopulations follow the population distribution from Table 1. For this figure, the Hotspot and Poor Treatment subpopulations were defined to have 100,000 individuals. Shaded areas indicating two thresholds for cost-effectiveness are drawn for reference, and points falling within a shaded region indicates that vaccinating the corresponding population or subpopulation is cost-effective or very cost-effective.

The estimated national average observed incidence of cholera in Bangladesh is 2.1 cases/1,000 population/year.^21^ Vaccinating the entire population is not cost-effective in this analysis as it would cost $3,113/DALY averted, and interventions need to cost < $2,250/DALY averted to be considered cost-effective in this setting (Table 2). However, over half of the population of Bangladesh lives in districts that are believed to be at high risk of cholera, with an estimated observed incidence of 3/1,000/year.^21^ Non-selective mass vaccination in these high-risk districts *would* be cost-effective, costing $2,156 per DALY averted, $825 per cholera case averted, and $54,980 per death averted (Table 2, Figure 1).

**Figure 2.**
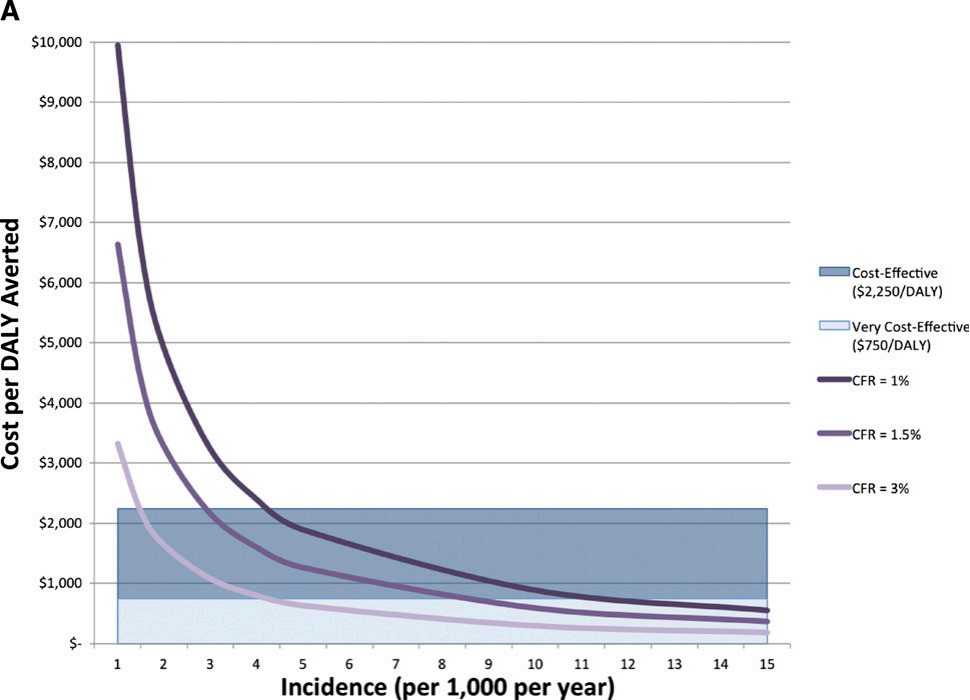

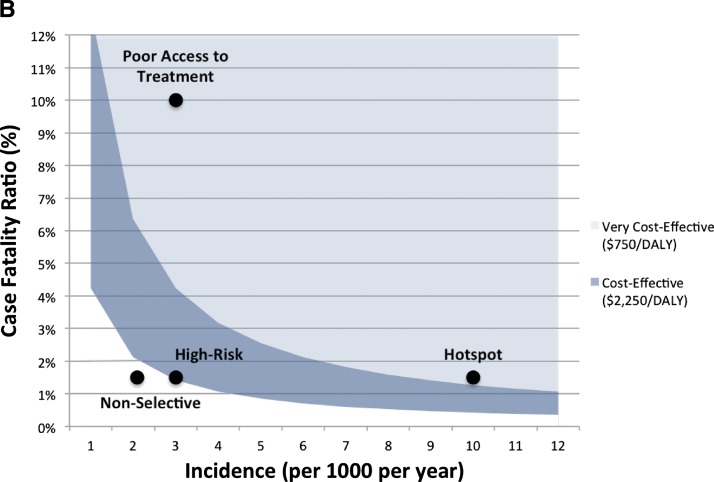
Sensitivity of cost-effectiveness to epidemiological parameters. (**A**) The cost per disability-adjusted life years (DALY) averted is sensitive to cholera incidence and case fatality ratio (CFR). The three curves plot the cost per DALY averted versus cholera incidence for three different CFRs, and two cost-effectiveness thresholds (shaded regions) are drawn for reference. (**B**) An alternative representation of the relationships in panel A is plotted, in which the relationship between cholera incidence and CFR is shown directly. Points show different combinations of cholera incidence and CFR, and those that lie above the thresholds are cost-effective or very cost-effective.

The cost-effectiveness of mass OCV vaccination is sensitive to cholera incidence, case fatality ratio, vaccine cost, vaccine duration, and vaccine efficacy (Figure 2). For a population with a CFR of 1.5% (the estimate for Bangladesh, Table 1), it is not cost-effective to vaccinate populations with an incidence < 2.89/1,000/year (Figure 2A). Although mass vaccination of the population of the high-risk districts in Bangladesh (3/1,000/year) may be cost-effective, any significant reduction in the estimate for the incidence or CFR of this population would result in a mass vaccination strategy that is NOT cost-effective (Figure 2B). Vaccine costs must be very low for OCV campaigns to be cost-effective when the vaccine efficacy is low and when the duration of immunity is short.

### Targeting children.

**Figure 3.**
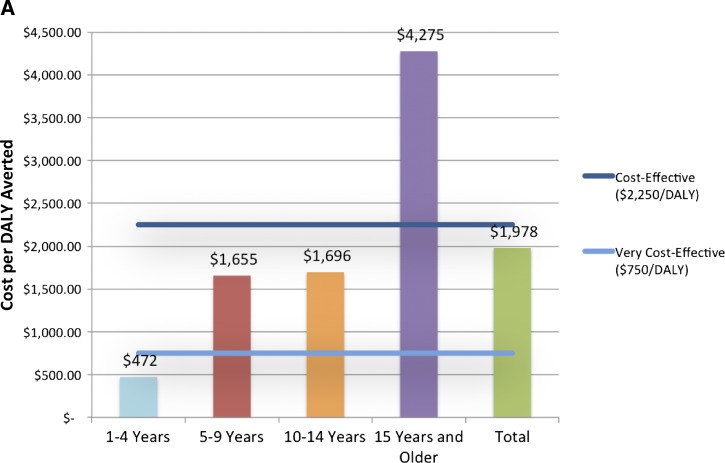

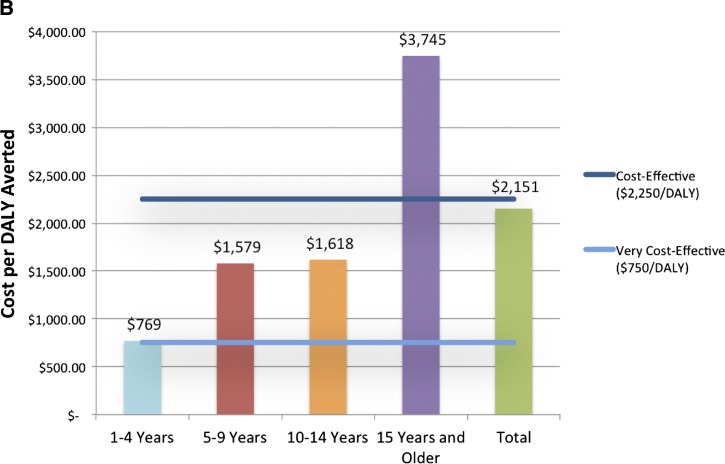
Cost-effectiveness of vaccinating different age groups. (**A**) Cost per disability-adjusted life years (DALY) averted when vaccinating members of each age group when the vaccine is 65% effective for all ages. The width of each bar is proportional to the population size of the corresponding age group. Bars that rise above a threshold would be considered cost-effective or very cost-effective. (**B**) Cost per DALY averted when the vaccine efficacy differs by each age group (42%, 68%, 68%, and 74% for toddlers, young children, older children, and adults, respectively).

Children in Bangladesh have a higher incidence of cholera than adults (Table 1). Figure 3 shows the cost-effectiveness of targeting different age groups for vaccination. Vaccinating children from 1 to 4 years of age in the high-risk districts costs < $500 per DALY averted and is very cost-effective (Figures 1 and 3A) when vaccine efficacy is 65%. The costs per DALY averted is higher in school-aged children (5–14 years of age), but it is still cost-effective to vaccinate these age groups ($1,678/DALY). Vaccinating adults (15 years and older) is not cost-effective in this scenario (Figure 3A). Vaccinating children 1–14 years of age, is more cost-effective ($1,034/DALY) than vaccinating adults ($4,275/DALY) because children have higher cholera incidence than adults and averting cholera-related deaths in children averts more years of life lost.^26^ However, some studies have found that OCV has lower efficacy in children than adults.^28^,^29^ Even when vaccine efficacy is only 42% among children 1–4 years of age, vaccinating this age group is still cost-effective ($769/DALY averted) (Figure 3B).

### Targeting cholera “hotspots.”

Geographic “hotspots,” or regions with much higher incidence than the surrounding areas, are likely to exist in epidemic and endemic cholera outbreaks. Prioritizing such areas over lower incidence surrounding regions can increase the cost-effectiveness of mass cholera vaccination. A disproportionate number of patients presenting to the International Center for Diarrheal Disease Research, Bangladesh (icddr,b) hospital in Dhaka live in the Mirpur neighborhood where hospitalization rates, probably a low estimate of total cholera incidence, can exceed 4/1,000/year.^4^,^42^,^43^ Targeting a spatial hotspot with a very high incidence of cholera (10/1,000/year) can be very cost-effective even in an endemic setting with a low CFR (Figures 1 and 2B). The cost per DALY averted in the hotspot is $592, the cost per case averted is $226, and the cost per death averted is $15,094 (Table 2).

### Targeting populations with relatively low access to care.

Individuals with poor access to safe water, sanitation, hygiene, and medical care are considered as a potential population for vaccination targeting. Vaccinating difficult-to-reach populations might double the delivery cost of a vaccination campaign,^9^,^31^–^34^ but cholera cases that occur in remote populations and do not receive proper treatment may suffer from a CFR of 10% or higher (Table 1).^21^,^27^ The cost per infection may be lower because of a reduced cost of treatment and lower rate of received treatment (Table 1).^44^ Under these assumptions, vaccinating such populations can be very cost-effective ($644/DALY averted, Figure 1). The cost per case averted is $1,684 and the cost per death averted is $16,844 (Table 2). Figure 2B illustrates the non-linear relationship between CFR and cost-effectiveness.

### Accounting for indirect protection from vaccination.

The analyses described previously assumed that only vaccinated individuals would benefit from mass vaccination and that the number of cases averted was proportional to the vaccination coverage, the vaccine's efficacy, and the incidence of cholera. In reality, mass vaccination could reduce the incidence of cholera therefore both vaccinated and unvaccinated individuals would have lower incidence (i.e., from indirect protection from vaccine). In a large individually randomized trial of OCV in rural Bangladesh, unvaccinated individuals in areas with higher vaccine coverage had lower cholera incidence than areas with lower coverage,^39^ which is evidence of indirect protection. A mathematical model of cholera transmission was calibrated using these results to extrapolate the effectiveness of mass vaccination at different coverage levels.^40^

**Figure 4.**
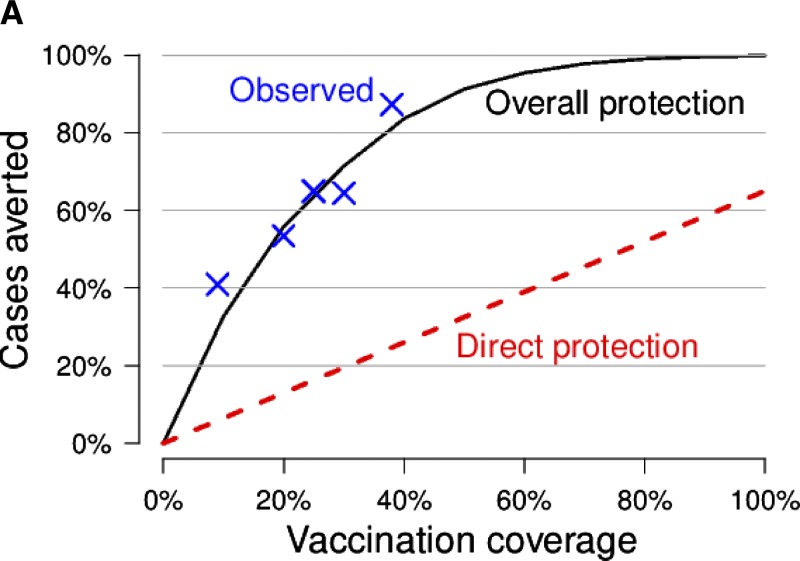

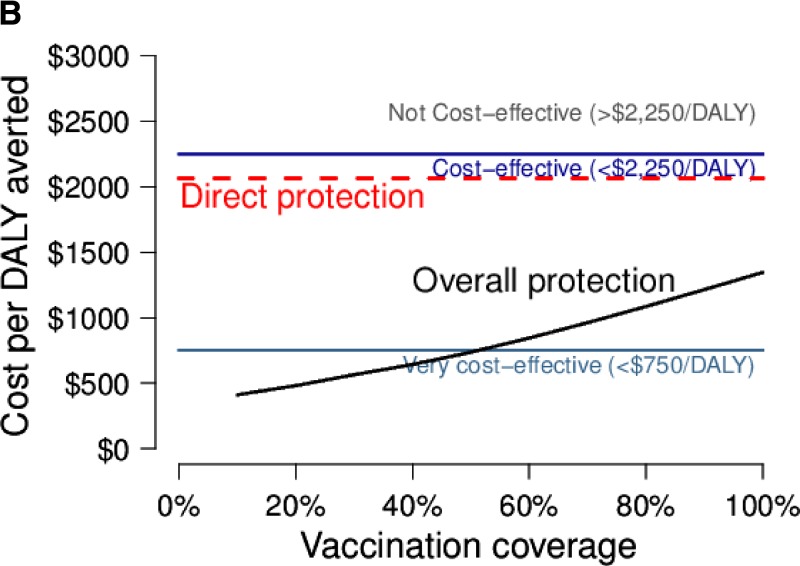
Including overall protection from mass vaccination. A mathematical model of cholera transmission was used to estimate the number of cases averted when a given fraction of the total population is vaccinated. (**A**) The model predicts that the fraction of cases averted by mass vaccination (black solid line) exceeds the estimates when only direct protection is assumed (red dashed line). The blue Xs indicate levels of protection observed in a cholera vaccine trial.^39^ In addition, the relationship between vaccine coverage and effectiveness is not linear when assuming overall protection, unlike direct protection. (**B**) Mass vaccination is more cost-effective when overall protection is considered compared with calculations that only account for direct protection.

When the dynamic transmission model is used to estimate the number of averted cases, rather than assuming the number of averted cases is proportional to vaccine coverage as assumed in the previous analyses, the proportion of cases averted rises sharply with increasing vaccine coverage then plateaus when coverage exceeds 70% (Figure 4A). Because a larger number of cases are averted when overall protection is considered, mass vaccination is more cost-effective (Figure 4B). The cost-effectiveness per person vaccinated is highest at low levels of coverage because each case averted would avert a larger amount of onward transmission. As coverage increases, the incidence of cholera decreases and each case averted by vaccination would therefore avert a smaller number of onward transmission events.

## Discussion

In this study, we have shown the potential use of a cost-effectiveness calculator (publicly available at stopcholera.org) to explore different mass vaccination strategies. This work has shown that the health and economic outcomes associated with the use of oral cholera vaccine varies by the strategy used in its deployment and can be cost-effective under certain conditions, particularly when high-risk subpopulations can be identified and targeted. Targeted efforts may dramatically improve the health and economic efficiency of cholera vaccination campaigns and such considerations should be a part of mass vaccination planning.

Vaccinating children in Bangladesh could be much more cost-effective than vaccinating the total population, a result consistent with previous OCV cost-effectiveness studies.^24^ In cholera-endemic regions, like Bangladesh, children may have higher rates of cholera than adults, probably caused by a lack of acquired immunity, and might have higher case fatality rates.^2^,^34^,^45^,^46^ Although several studies have shown that OCV efficacy and duration of protection may be lower in children than in adults, mass cholera vaccination of children between 1 and 4 years of age can still be cost-effective if cholera incidence is sufficiently high.^28^,^29^

Vaccinating children might also be more logistically feasible than vaccinating adults. Many countries have existing infrastructure to deliver vaccinations to infants and school children but as Shanchol is not recommended for use in children < 1 year of age, OCV might be best delivered in accompaniment with regular or catch-up immunization days, fixed sites, through schools, or alongside other Enhanced Program on Immunization (EPI) campaigns.^4^,^21^,^47^ Ensuring that adults receive both doses of a 2-dose vaccine poses logistical challenges that may require the development of new or expansion of existing programs that could conceivably increase the cost of vaccinating adults.

Geographic hotspots and areas with elevated cholera incidence have been identified in cholera-endemic regions and cholera outbreaks.^4^,^27^,^42^–^44^ From both a public health and an economic perspective, targeted vaccination reaching populations that are at high risk of infection is highly advantageous. This type of targeting requires detailed spatio-temporal cholera incidence data that are not always available, as countries often report only nationwide incidence rates. Efforts to strengthen cholera surveillance capacity in Africa, such as Africhol may provide the ability to translate some of these findings from Bangladesh to African contexts.^48^

Cost-effectiveness analyses that use DALYs averted as the primary metric are sensitive to changes in disease-associated mortality rates, therefore OCV may be very economically efficient if targeted to individuals with a low likelihood of receiving treatment. The widespread availability and use of oral and intravenous rehydration therapy for cholera has dramatically reduced the CFR associated with cholera from 50% to < 1% in properly treated patients but mortality can be significantly higher in epidemic settings.^10^,^35^,^49^–^52^ The incidence estimates in this analysis are of cases that seek treatment; vaccination would also reduce the incidence of cholera in those that do not seek treatment, therefore the DALYs averted in this analysis are likely underestimates of the overall number. As cholera is a disease associated with poor governance, poverty, and social inequity, targeted vaccination reaching the most vulnerable and at-risk populations is favorable from economic, health, and equity viewpoints.^53^,^54^

Large-scale vaccination trials have shown that indirect protection from mass cholera vaccination can be substantial.^39^,^55^ The VICE tool is not appropriate for evaluating long-term effects of vaccination on health outcomes and transmission dynamics such as waning immunity, natural immunity and boosting, and cholera elimination and cannot predict indirect effects of vaccine protection on the unvaccinated population but mathematical models have been used to estimate indirect protection of mass cholera vaccination.^40^ However, mathematical models need to be calibrated to the epidemiology of a specific time and place, and their results are difficult to generalize to other scenarios. Therefore, we did integrate mathematical modeling into the general-purpose tool, which produces conservative estimates of cost-effectiveness by assuming only direct protection. We can make general, but non-quantitative, conclusions about overall protection from mass vaccination. The overall protection (the combination of direct and indirect effects) from mass vaccination is highest at intermediate coverage levels, and plateaus once a critical vaccination fraction is reached. Including indirect protection may be required to show that mass cholera vaccination can be cost-effective in some populations.^17^,^24^

In recent estimates, OCV compares relatively favorably with typhoid vaccine ($179–4863/DALY averted) but is much less cost-effective than rotavirus vaccine ($22–279/DALY averted), probably because of the widespread prevalence and limited age range of rotavirus infection.^56^ The cross-protective effects of OCV against other diarrheal diseases was not considered in this analysis but would make vaccination more cost-effective.^57^ With increased production and experience with its delivery, the cost for purchase and program costs for OCV should decrease. In fact, the analyses presented here show that mass cholera vaccination is more cost-effective than a few years ago,^21^ with the recent prequalification of a less expensive vaccine, expanded financial leveraging of vaccine production and deployment, the increased life expectancy of the population of Bangladesh, and the increased GDP of Bangladesh.^38^,^58^,^59^ We expect some of these trends to continue, making mass cholera vaccination even more cost-effective in the future.

The results presented here are largely based on Bangladesh-specific parameters, therefore they should not be considered optimal across different settings. Although we used the same methodology for calculating cost-effectiveness as a previous studies of the cost-effectiveness of cholera vaccination in Bangladesh, we found that mass cholera vaccination in high-risk districts of Bangladesh is cost-effective while previous studies found mass vaccination of the general population was not cost-effective unless indirect protection was considered.^21^,^24^ The differences between our results and those reported by others highlight that DALY-based cost-effectiveness analyses are highly sensitive to targeted vaccination based on demographic, epidemiological, and economic characteristics and highlight the importance of context-specific parameters, which could be further explored using sensitivity analyses.

Cost-effectiveness alone may not be sufficient to introduce vaccines in developing countries and often must be considered with additional budgetary, logistical, and political factors.^20^ We have shown that oral cholera vaccination can be cost-effective in Bangladesh, an endemic setting, and that this cost-effectiveness model may be a valuable tool for health officials, funders, governments, and other policy experts to make difficult decisions about the use of oral cholera vaccine as part of a comprehensive approach to cholera control. The model allows for comparisons between populations with different disease epidemiology, is flexible to changes in costs and economic parameters, and is applicable to vaccination campaigns for other diseases besides cholera. As oral cholera vaccine becomes more widely available and used in the next decade, these types of economic evidence and cost-effectiveness analysis will play an increasingly valuable role in decisions to use the vaccine.

## Figures and Tables

**Table 1:** Cost-effectiveness model parameters

Parameters	Non-selective scenario	Age-specific, “high-risk” districts	Geographic hotspot	Poor access to care
Age distribution		10% (1–4 years)		
		15% (5–9 years)		
		13% (10–14 years)		
		62%^26^ (15+ years)		
Observed incidence per 1,000 per year	National average: 2.1^21^“High-risk” districts: 3^21^	11 (1–4 years) 3.5 (5–14 years) 1.7 (15+ years)^21^	10^27^	3^21^
Case fatality ratio	1.5%^21^	1.5%^21^	1.5%^21^	10%^27^
Life expectancy at age of infection (years)	51^26^	71 (1–4 years)	51^26^	51^26^
		68 (5–9 years)		
		63 (10–14 years)		
		41 (15+ years)^26^		
Vaccine efficacy (direct protection, Shanchol)	65%^28^,^29^	65% (Overall)	65%^28^,^29^	65%^28^,^29^
		42% (1–4 years)		
		68% (5–14 years)		
		74% (15+ years)^28^		
Duration of immunity (years, Shanchol)	3^28^,^29^	3^28^,^29^	3^28^,^29^	3^28^,^29^
Total cost of vaccine purchasing and delivery (2 doses, Shanchol)	$5^4^,^30^	$5^4^,^30^	$5^4^,^30^	$10^9^,^31^–^33^
Cost of infection (total: public + private)	$30^21^,^23^,^24^	$30^21^,^23^,^24^	$30^21^,^23^,^24^	$25^23^
Illness duration (days)	4^24^,^34^,^35^	4^24^,^34^,^35^	4^24^,^34^,^35^	4^24^,^34^,^35^
Disability weight (duration of illness only)	0.202^36^	0.202^36^	0.202^36^	0.202^36^
Annual discount rate	3%^15^	3%^15^	3%^15^	3%^15^
GDP/Capita (2012)	$750^37^	$750^37^	$750^37^	$750^37^

**Table 2:** Estimated cost-effectiveness of cholera vaccination over 3 years

	Non-selective countrywide (2.1/1,000/year)	Non-selective high-risk districts (3/1,000/year)	Children < 15 years targeted	Children < 15 years targeted, (42% 1–4; 68% 5–14)	Hotspot targeted-(10/1,000/year)	Poor access to treatment population targeted-(10% CFR)
No. vaccinated per case averted	244	171	94	113	51	171
Cost/case averted	$1,191	$825	$440	$533	$226	$1,684
No. vaccinated per death averted	16,280	11,364	6,230	7,501	3,448	1,709
Cost/death averted	$79,400	$54,980	$29,365	$35,507	$15,094	$16,844
Cost/DALY averted	$3,113	$2,156	$1,034	$1,256	$592	$664

CFR = case fatality ratio; DALY = disability-adjusted life years.
